# Epidemiology of chronic kidney disease in northern region of Senegal: a community-based study in 2012

**DOI:** 10.11604/pamj.2014.18.307.3636

**Published:** 2014-08-16

**Authors:** Sidy Mohamed Seck, Dominique Doupa, Lamine Guéye, Charles Abdou Dia

**Affiliations:** 1 Internal Medicine and Nephrology Department, Faculty of Health Sciences, University Gaston Berger, Senegal; 2 Biochemistry Department, Faculty of Health Sciences, University Gaston Berger, Senegal; 3 Neurology Department, Faculty of Health Sciences, University Gaston Berger, Senegal; 4 District Hospital of Saint-Louis, Senegal

**Keywords:** Chronic kidney disease, epidemiology, population, Senegal

## Abstract

**Introduction:**

Chronic kidney disease (CKD) is an emerging worldwide epidemic but few data are available in African populations. We aimed to assess prevalence of CKD in adult populations of Saint-Louis (northern Senegal).

**Methods:**

In a population-based survey between January and May 2012, we included 1,037 adults aged =18 years living in Saint-Louis. Socio-demographical, clinical and biological data were collected during household visits. Serum creatinine was measured by Jaffé method. We estimated glomerular filtration rate (eGFR) using the 4-variables MDRD equation and CKD was defined by eGFR < 60 mL/min/1.73m^2^ and/or albuminuria > 1g/L. A multivariate logistic regression was performed to identify factors associated with CKD.

**Results:**

Mean participants’ age was 47.9 ±16.9 years (18-87) and sex-ratio was 0.52. Majority of participants lived in urban areas (55.3% rural) and had school education (65.6%). Overall prevalences of hypertension, diabetes and obesity were 39.1%, 12.7% and 23.4% respectively. Prevalence of CKD was 4.9% (95% CI= 3.5 – 6.2) and 0.9% had GFR < 30 mL/min/1.73m^2^. Albuminuria >1g/l was found in 3.5% of people. CKD was significantly more frequent among hypertensive patients compared to normotensive participants. Only 23% of patients were aware of their disease before the survey. After multivariate logistic analysis, presence of CKD was significantly associated with hypertension (OR=1.12, p= 0.02) and age (OR=1.03, p= 0.02).

**Conclusion:**

CKD is frequent in adult population living Northern Senegal. Main associated factors are hypertension and age. Prevention strategy is urgently needed to raise awareness and promote CKD detection and early treatment in both urban and rural areas.

## Introduction

Chronic kidney disease (CKD) represents one of the greatest public health challenges in the 21^st^ century and is associated with an important cardiovascular morbidity and mortality [1, 2]. It has a major impact on healthcare costs and world productivity particularly in low-income countries where the young people are the most concerned [3]. Early detection combined with an adequate management of patients are the best strategy to fight this disease. In Africa, though we assist to a rising awareness of health authorities about CKD burden, prevention and management programs are still difficult to elaborate because of the scarcity of epidemiological data at population level [3, 4]. Most of current data come from nephrology departments in specialized hospitals in big cities and there is very little information in rural populations [5].

This study aimed to assess prevalence of chronic kidney disease (CKD) in urban and rural areas of Saint-Louis, northern region of Senegal.

## Methods

**Study design:** We performed a community-based cross-sectional survey in Saint-Louis (northern region of Senegal). All individuals aged =18 years and living in Saint-Louis since at least 3 months were eligible to participate in the study.

**Sampling procedure:** A two-stage cluster sampling method was used to select a representative sample of adults living in urban and rural areas of Saint-Louis. We firstly selected 17 localities as clusters (9 urban areas and 8 rural areas). Then, we randomly took a number of households proportionally to population size of each locality (data available from the National Agency of Statistics and Demography). From each household a maximum of two participants were recruited.

Considering a a-error of 0.05 and a power ^6; of 80%, the required sample size was 855 individuals and we added a 20% attrition rate to get a final sample of 1026 participants.

**Data collection:** Data were collected on-site during house-to-house visits that were conducted between 7a.m. and 12 a.m. or at the nearest health centre when patients did not live far away from this facility.

A modified version of the WHO STEPS wise questionnaire was pre-tested and validated before its use to collect data. Researchers assisted by medical students, trained nurse practitioners, and community health workers had to fill the data collection form, to document the socio-demographic status (age, sex, marital status, education, profession and education), personal and family health history (regarding particularly hypertension, diabetes, stroke, heart and kidney disease), and lifestyle (nutritional habits, physical activity, smoking and alcohol consumption) of each participant. History of nephrotoxic medications (non-steroidal anti-inflammatory drugs and traditional herbs) was also assessed. Anthropometric measurements (weight, height, waist and hip circumference) were performed using standard methods and calibrated devices. Serum total cholesterol, LDL cholesterol, HDL cholesterol, and Triglycerides were measured with colorimetric method. Obesity was defined using International Diabetes Foundation cut-offs [6]. Blood pressure was measured twice at five minutes intervals by a semiautomatic sphygmomanometer and the mean of the two readings was calculated. If the difference between the readings was greater than 10 mm Hg, a third measurement was performed.

Hypertension was defined as a systolic blood pressure of 140 mm Hg or more, diastolic blood pressure of 90 mm Hg, any prescription of antihypertensive medication in the past two weeks, or any self-reported history of hypertension [7]. Fasting blood glucose (FBG) was measured with a glucose oxidase method. Diabetes was defined as FBG = 1.26 mg/dL, by prescription of hypoglycemic agents despite fasting plasma glucose, or any self-reported history of diabetes [6].

Physical inactivity was defined as less than 30 minutes of moderate activity per week or less than 20 minutes of vigorous activity three times per week, or the equivalent.

Serum creatinine was measured with Jaffe's kinetic method and glomerular filtration rate (GFR) was estimated with the MDRD equation [8]. Urinary albumin excretion (UAE) was first screened using strips and positive cases were confirmed by a quantitative dosage in the 24 hours urine output. CKD was defined according to National Kidney Foundation classification [9].

**Ethical issues:** The study was approved by the National Committee for Ethics in Health Research. A free consent form had to be signed by participants to give their approval before data collection. All participants were personally informed of their screening results and those with abnormal values were referred to a specialist for further exploration and treatment.

**Statistical analysis:** Statistical analyses were performed with STATA 12.0 (Stata Corp, TX, USA). Continuous variables were presented as mean ± standard deviation and categorical variables as percentage. Comparison of proportions and means were done using Pearson's Chi-square test or Student's t-test as appropriated. Multivariate regression analysis was used to identify clinical and biological parameters associated with CKD. A p-value =0.05 was considered as statistically significant.

## Results

A total of 1047 individuals were interviewed and 1037 had a complete questionnaire and were kept in the study (response rate of 99%).

Fifty three percent of them lived in urban areas and 60% were female. Table 1 presents demographical, clinical and biological characteristics of participants according to their living areas. Compared to rural population, participants from urban cities were older, more educated, more active, and more likely to be women. One participant out of ten reported a relative with a history of renal disease. Cardiovascular risk factors such as hypertension, diabetes and obesity were frequent among the whole population with a significantly higher prevalence in individuals living in urban areas (Table 1).

**Table 1:** Demographical and clinical characteristics of participants. (Data are expressed as mean ±standard deviation or number and percentage)

	All participants (n=1036)	Urban areas (n=578)	Rural areas (n=458)	p-value
**Age (years)**	48.0 ±16.9 (18-87)	51.6 ±15.7	43.5 ±17.2	0.001
**Age group**				0.001
18-34 years	25.6%	16.0%	37.8%	
35-49 years	25.3%	26.1%	24.2%	
50-60 years	23.6%	28.0%	18.1%	
>60 years	25.5%	29.9%	19.9%	
School education	60.7%	63.4%	55.6%	0.030
Familial history of renal disease	10.4%	5.4%	15.0%	0.003
Tobacco use	4.2%	5.2%	2.8%	0.094
Alcohol use	4.5%	1.5%	8.5%	<0.001
Physical inactivity	58.1%	55.3%	61.7%	0.047
Hypertension	39.1%	43.3%	33.8%	0.002
Diabetes (FBG≥1.26g/l)	12.7%	14.6%	10.3%	0.038
BMI (kg/m^2^)	26.3 ±6.8	27.9 ±7.3	24.3 ±5.5	<0.001
Waist circumference	90.6 ±16.1	94.4 ±15.6	86.0 ±15.6	<0.001
Obesity (BMI≥30 kg/m^2^)	23.4%	33.8%	10.2%	<0.001
Cholesterol (g/L)	2.18 ±0.49	2.25 ±0.54	2.10 ±0.44	<0.001
SCr (mg/dl)	1.04 ±0.44	1.01 ±0.47	1.06 ±0.40	0.032
eGFR (ml/min)	90.6 ±23.8	89.7 ±22.8	91.7 ±24.9	0.203
Albuminuria>1g/l	5.3%	4.3%	6.6%	0.178

BMI= body mass index; FBG= fasting blood glucose; SCr= serum creatinine; eGFR= estimated glomerular filtration rate according to 4-variables MDRD equation.

**Figure 1:**
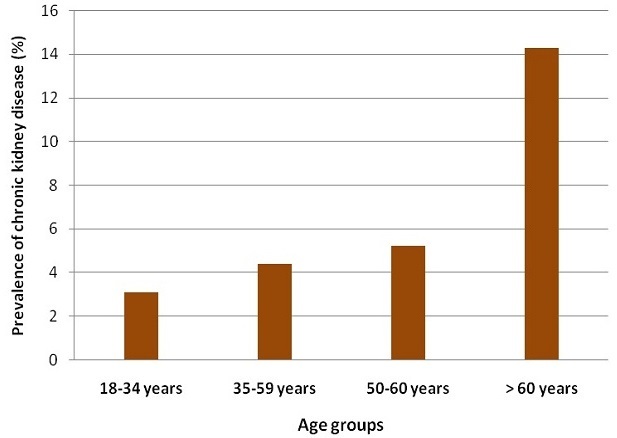
Prevalence of chronic kidney disease according to age groups

Overall adjusted prevalence of CKD in the total population was 6.1% (95%confidence interval between 4.7% and 7.6%) with 3.5% of individuals presenting with albuminuria > 1g/L. Chronic kidney disease prevalence in women (5.7%) was lower than in men (6.9%). A linear increase with age was found in CKD prevalence (p for trend = 0.03). In the subgroup of people aged > 60 years, 14.3% presented CKD (Figure 1).

**Figure 2:**
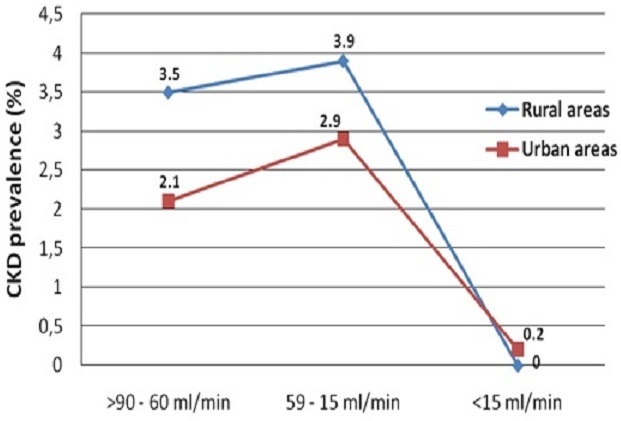
Level of glomerular filtration rate in urban and rural populations

Surprisingly, people living in rural areas showed a similar prevalence of CKD compared to those in urban cities (respectively 7.6% and 5.0%, p = 0.08) even though early stages 1 and 2 were more frequent in urban areas (Figure 2). Eighty three percent of people with CKD in rural areas and 62.7% in urban areas were not aware of their disease before the study.

In the univariate analysis, CKD was significantly associated with hypertension, obesity, physical activity, familial history of kidney disease and age group (Table 2).

**Table 2:** Association between chronic kidney disease and socio-demographical and clinical factors (univariate analysis)

	Odds Ratio	[95% Confidence Interval]	p-value
Age group	1.08	1.02 - 1.51	0.02
Family history of renal disease	1.12	0.76 - 1.45	0.05
School education	0.99	0.76 - 2.54	0.17
Physical inactivity	0.65	0.57 - 0.99	0.05
Hypertension	2.15	1.61 - 4.36	0.03
Diabetes	1.22	0.33 - 40.74	0.63
Obesity	1.33	1.05 - 1.98	0.04

BP= blood pressure; eGFR= estimated glomerular filtration rate according to MDRD equation

After multivariate regression analysis, only age and high blood pressure remained significantly associated with CKD while diabetes, familial history and obesity failed to reach statistical significance (Table 3).

**Table 3:** Multivariate regression analysis of factors associated with chronic kidney disease

	Odds Ratio	[95% Confidence Interval]	p-value
Age group	1.03	1.00 - 1.06	0.018
Hypertension	1.12	1.02 - 1.23	0.020
Diabetes	0.80	0.25 - 2.51	0.703
Family history of renal disease	1.43	0.38 - 5.33	0.597
Obesity	0.77	0.24 - 2.46	0.662
Urban (*vs* rural) residents	0.34	0.13 - 0.87	0.024
School education	0.85	0.33 - 2.19	0.741

N =324 Pseudo R^2^ = 0.106

## Discussion

This study is the first one that assessed in a sample of Senegalese population the burden of CKD particularly for the early stages (1 and 2) which are often not seen by specialists at hospital [10]. Globally, prevalence of CKD is estimated to 10% but there are many variations between countries and within the same countries [11].

In sub-Saharan Africa, epidemiology of CKD in general population is difficult to estimate because of possible confounding due to heterogeneity of study populations, design and methods used to define CKD (1). Community-based studies are very scarce and most of previously reported data come from hospital records [2, 3]. CKD prevalence in our population was lower than previous reports from community-based studies in Ghana (13.2%) [5] and Kinshasa (12.4%) [12]. This study also confirmed the increase of CKD prevalence with age already described in other populations [1, 3]. However, the level of increase was less important compared to US population where more than 20% of people aged >60 years have CKD [13].

As expected, our results found less than one quarter of CKD patients who were aware of their disease particularly in rural areas were education level is lower and people do not easy access to healthcare services. Similar low rates of awareness had been commonly reported in other populations [14] and even health workers [15]. More efforts are needed to sensitize governments and populations about the disease burden [1].

CKD risk factors like high blood pressure, diabetes, obesity and abdominal obesity were frequent among participants [2, 11]. However, only age and hypertension independently correlated with CKD while other risk factors. Some risk factors like infections, genetic predispositions and environmental or herbal toxins were not evaluated in this study but they might play a prominent role in African populations [16, 17].

Interestingly, one CKD patient over nine in our study was detected at early stages where therapeutic interventions are most efficient [2, 10]. In developing countries, diagnosis of patients at ESRD has a limited interest because a few proportions of them will have access to renal replacement therapy with a lot of difficulties [18].

In order to achieve long-term reduction of CKD morbidity and mortality, early detection and prevention at population level are the best cost-effective strategy because conservative treatment has limited public health impact [2, 4, 19]. Developed countries spend 2-3% of their health expenditures for management of ESRD patients who represent less than 0.03% of patients [20]. The situation is probably worse in low-income countries where healthcare expenditure is often very low and the majority of ESRD patients die because of dialysis inaccessibility [3].

This study has many limits due to its cross-sectional design, inferences about causality or direction of association should be made with caution.

Nevertheless, it gives an insight on the epidemic of CKD in the northern region of Senegal. More longitudinal studies are urgently needed to produce reliable data about CKD incidence, prevalence, and risk and progression factors in sub-Saharan African populations [1, 3]. Integrating CKD in other chronic diseases programs could be an interesting approach to increase coverage of screening and awareness in populations. However, mass screening might not be beneficial from a public health perspective because of potential overdiagnosis of cases that will undergo unnecessary investigation or referral to secondary care and the high cost for the healthcare system [21]. Also, cost-effectiveness of current preventive interventions should be assessed in relation to the local socio-economic context particularly in rural areas [1, 4].

## Conclusion

This study reveals a high prevalence of chronic kidney disease in northern region of Senegal. Urban and rural areas are similarly concerned and awareness rate is very low among populations. Early asymptomatic stages are the most frequent and associated risk factors are hypertension and aging. Integration of CKD screening to routine general medicine visits would improve early detection and management of patients with renal diseases.

## References

[1] Jha V, Garcia-Garcia G, Iseki K, Li Z, Naicker S, Plattner B, Saran R, Wang AYM, Yang CW (2013). Chronic kidney disease: global dimension and perspectives. Lancet..

[2] Couser WG, Remuzzi G, Mendis S, Tonelli M (2011). The contibution of chronic kidney disease to the global burden of major noncommunicable diseases. Kidney Int..

[3] Abegunde D, Mathers CD, Adam T, Ortegon M, Strong K (2007). The burden and costs of chronic diseases in low-income and middle-income countries. Lancet..

[4] Hallan SI, Stevens P (2010). Screening for chronic kidney disease: which strategy. J Nephrol..

[5] Eastwood JB, Kerry SM, Plange-Rhule J, Micah FB, Antwi S, Boa FG, Banerjee D, Cappuccio FP (2010). Assessment of GFR by four methods in adults in Ashanti, Ghana: the need for an eGFR equation for lean African populations. Nephrol Dial Transplant..

[6] The IDF worldwide definition of the metabolic syndrome. The International Diabetes Foundation. http://www.idf.org/webdata/docs/IDF_Meta_def_final.pdf.

[7] Chobanian AV, Bakris GL, Black HR, Cushman WC, Green LA, Izzo JL, Jones DW (2003). The Seventh Report of the Joint National Committee on Prevention, Detection, Evaluation, and Treatment of High Blood Pressure: the JNC 7 report. JAMA..

[8] Levey AS, Bosch JP, Lewis JB, Greene T, Rogers N, Roth D (1999). A more accurate method to estimate glomerular filtration rate from serum creatinine: a new prediction equation. Ann Intern Med..

[9] Levey AS, Coresh J, Balk E, Kausz AT, Levin A, Steffes MW, Hogg RJ, Perrone RD, Lau J, Eknoyan G (2003). National Kidney Foundation Practice Guidelines for Chronic Kidney Disease: Evaluation, Classification, and Stratification. Ann Intern Med..

[10] Perico N, Bravo RF, De Leon FR, Remuzzi G (2009). Screening for chronic kidney disease in emerging countries: feasibility and hurdles. Nephrol Dial Transplant..

[11] James MT, Hemmelgarn BR, Tonelli M (2010). Early recognition and prevention of chronic kidney disease. Lancet..

[12] Sumaili EK, Krzesinski JM, Cohen EP, Nseka NM (2010). Épidémiologie de la maladie rénale chronique en République Démocratique du Congo: une revue synthétique des études de Kinshasa, la capitale. Nephrol Ther..

[13] Coresh J, Selvin E, Stevens LA (2007). Prevalence of chronic kidney disease in the United States. JAMA..

[14] Plantinga LC, Bouleware LE, Coresh J (2008). Patient awareness of chronic kidney diseases: trends and predictors. Arch Intern Med..

[15] Minutolo R, De Nicola L, Mazzaglia G (2008). Detection and awareness of moderate to advanced CKD by primary care practitioners: a cross-sectional study from Italy. Am J Kidney Dis..

[16] Kanji Z, Powe CE, Wenger JE (2011). Genetic variations in APOL1 associates with younger ages at hemodialysis initiation. J Am Soc Nephrol..

[17] Jha V, Rathi M (2008). Natural medicines causing acute kidney injury. Semin Nephrol..

[18] White SL, Chadban SJ, Jan S, Chapman JR, Cass A (2008). How can we achieve global equity in provision of renal replacement therapy. Bull World Health Organ..

[19] Black C, Sharma P, Scotland G, McCullough K, McGurn D, Robertson L (2010). Early referral strategies for management of people with markers of renal disease: a systematic review of the evidence of clinical effectiveness, cost-effectiveness and economic analysis. Health Technol Assess..

[20] Levey AS, Atkins R, Coresh J (2007). Chronic kidney disease as a global public health problem: approaches and initiatives- a position statement from Kidney Disease Improving Global Outcomes. Kidney Int..

[21] Taal MW (2012). Screening for Chronic Kidney Disease: Preventing Harm or Harming the Healthy. PLoS Med..

